# Tetra­kis(μ-4-methyl­benzoato-κ^2^ *O*:*O*′)bis­[(isonicotinamide-κ*N*)copper(II)]

**DOI:** 10.1107/S1600536810006513

**Published:** 2010-02-27

**Authors:** Hacali Necefoğlu, Efdal Çimen, Barış Tercan, Hakan Dal, Tuncer Hökelek

**Affiliations:** a Department of Chemistry, Kafkas University, 36100 Kars, Turkey; b Department of Physics, Karabük University, 78050 Karabük, Turkey; c Department of Chemistry, Faculty of Science, Anadolu University, 26470 Yenibağlar, Eskişehir, Turkey; d Department of Physics, Hacettepe University, 06800 Beytepe, Ankara, Turkey

## Abstract

In the title centrosymmetric binuclear complex, [Cu_2_(C_8_H_7_O_2_)_4_(C_6_H_6_N_2_O)_2_], the Cu atoms [Cu⋯Cu = 2.6375 (6) Å] are bridged by four 4-methyl­benzoate (PMB) ligands. The four nearest O atoms around each Cu^II^ ion form a distorted square-planar arrangement, and the distorted square-pyramidal coordination is completed by the pyridine N atom of the isonicotinamide (INA) ligand. Each Cu^II^ ion is displaced by 0.2633 (1) Å from the plane of the four O atoms, with an average Cu—O distance of 1.974 (2) Å. The dihedral angles between carboxyl­ate groups and the adjacent benzene rings are 7.88 (19) and 9.68 (10)°, while the benzene rings are oriented at a dihedral angle of 85.90 (9)°. The pyridine ring is oriented at dihedral angles of 8.59 (7) and 83.89 (9)° with respect to the benzene rings. In the crystal structure, inter­molecular N—H⋯O hydrogen bonds link the mol­ecules into a three-dimensional network. π–π contacts between the benzene rings and between the pyridine and benzene rings, [centroid–centroid distances = 3.563 (2) and 3.484 (2) Å, respectively] may further stabilize the crystal structure.

## Related literature

For niacin, see: Krishnamachari (1974 ▶), and for the nicotinic acid derivative *N*,*N*-diethyl­nicotinamide, see: Bigoli *et al.* (1972 ▶). For related structures, see: Hökelek *et al.* (1995 ▶, 2009*a* ▶,*b* ▶,*c* ▶); Speier & Fulop (1989 ▶); Usubaliev *et al.* (1980 ▶).
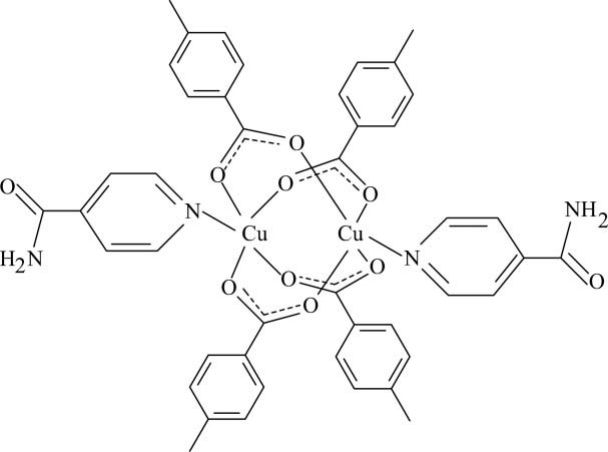


## Experimental

### 

#### Crystal data

[Cu_2_(C_8_H_7_O_2_)_4_(C_6_H_6_N_2_O)_2_]
*M* *_r_* = 911.88
Monoclinic, 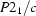

*a* = 11.2305 (2) Å
*b* = 23.4691 (4) Å
*c* = 8.0087 (1) Å
β = 102.128 (1)°
*V* = 2063.74 (6) Å^3^
*Z* = 2
Mo *K*α radiation
μ = 1.10 mm^−1^
*T* = 101 K
0.30 × 0.24 × 0.14 mm

#### Data collection

Bruker Kappa APEXII CCD area-detector diffractometer
Absorption correction: multi-scan (*SADABS*; Bruker, 2005 ▶) *T*_min_ = 0.735, *T*_max_ = 0.862
20056 measured reflections
5101 independent reflections
3629 reflections with *I* > 2σ(*I*)
*R*_int_ = 0.062

#### Refinement

*R*[*F* ^2^ > 2σ(*F* ^2^)] = 0.043
*wR*(*F* ^2^) = 0.087
*S* = 1.01
5101 reflections
281 parameters
H atoms treated by a mixture of independent and constrained refinement
Δρ_max_ = 0.47 e Å^−3^
Δρ_min_ = −0.70 e Å^−3^

### 

Data collection: *APEX2* (Bruker, 2007 ▶); cell refinement: *SAINT* (Bruker, 2007 ▶); data reduction: *SAINT*; program(s) used to solve structure: *SHELXS97* (Sheldrick, 2008 ▶); program(s) used to refine structure: *SHELXL97* (Sheldrick, 2008 ▶); molecular graphics: *ORTEP-3 for Windows* (Farrugia, 1997 ▶); software used to prepare material for publication: *WinGX* (Farrugia, 1999 ▶) and *PLATON* (Spek, 2009 ▶).

## Supplementary Material

Crystal structure: contains datablocks I, global. DOI: 10.1107/S1600536810006513/xu2729sup1.cif

Structure factors: contains datablocks I. DOI: 10.1107/S1600536810006513/xu2729Isup2.hkl

Additional supplementary materials:  crystallographic information; 3D view; checkCIF report

## Figures and Tables

**Table 1:** Selected bond lengths (Å)

Cu1—O1	1.9733 (18)
Cu1—O2^i^	1.9703 (18)
Cu1—O3	1.9687 (18)
Cu1—O4	1.9836 (18)
Cu1—N1	2.161 (2)

Symmetry code: (i) 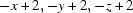.

**Table 2:** Hydrogen-bond geometry (Å, °)

*D*—H⋯*A*	*D*—H	H⋯*A*	*D*⋯*A*	*D*—H⋯*A*
N2—H2*A*⋯O5^ii^	0.89 (3)	2.11 (3)	2.984 (3)	169 (3)

Symmetry code: (ii) 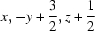.

**Fig. 1.**
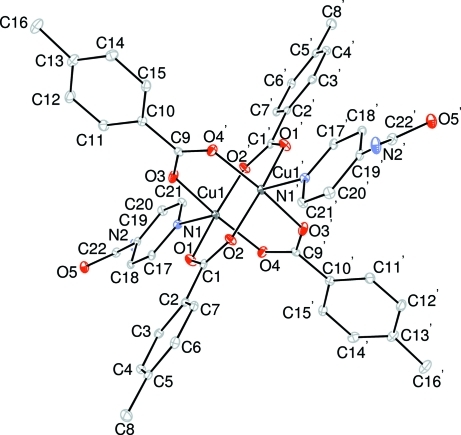
The molecular structure of the title molecule with the atom-numbering scheme. Displacement ellipsoids are drawn at the 20% probability level. Primed atoms are generated by the symmetry operator: (') 2-x, 2-y, 2-z.

## supplementary crystallographic information

### Comment

As a part of our ongoing investigation on transition metal complexes of nicotinamide (NA), one form of niacin (Krishnamachari, 1974), and/or the nicotinic acid derivative *N*,*N*-diethylnicotinamide (DENA), an important respiratory stimulant (Bigoli *et al.*, 1972), the title compound was synthesized and its crystal structure is reported herein.

The title compound is a binuclear compound, consisting of two INA and four 4-methylbenzoate (PMB) ligands. The crystal structures of similar complexes of Cu^2+^ and Zn^2+^ ions, [Cu(C_6_H_5_COO)_2_(C_5_H_5_N)]_2_ (Usubaliev *et al.*, 1980); [Cu(C_6_H_5_CO_2_)_2_(Py)]_2_ (Speier & Fulop, 1989), [Cu_2_(C_6_H_5_COO)_4_(C_10_H_14_N_2_O)_2_] (Hökelek *et al.*, 1995), [Zn_2_(C_11_H_14_NO_2_)_4_(C_10_H_14_N_2_O)_2_] (Hökelek *et al.*, 2009a), [Zn_2_(C_8_H_8_NO_2_)_4_(C_10_H_14_N_2_O)_2_].2H_2_O (Hökelek *et al.*, 2009b) and [Zn_2_(C_9_H_10_NO_2_)_4_(C_10_H_14_N_2_O)_2_] (Hökelek *et al.*, 2009c) have also been reported. In these structures, the benzoate ion acts as a bidentate ligand.

The title dimeric complex, [Cu_2_(PMB)_4_(INA)_2_], has a centre of symmetry and two Cu^II^ ions are surrounded by four PMB groups and two INA ligands (Fig. 1). The INA ligands are coordinated to Cu^II^ ions through pyridine N atoms only. The PMB groups act as bridging ligands. The Cu···Cu' distance is 2.6375 (6) Å. The average Cu—O distance is 1.9740 (18) Å (Table 1), and four O atoms of the bridging PMB ligands around each Cu^II^ ion form a distorted square plane. The Cu^II^ ion lies 0.2633 (1) Å below the least-squares plane. The average O—Cu—O bond angle is 89.39 (8)°. A distorted square-pyramidal arrangement around each Cu^II^ ion is completed by the pyridine N atom of INA ligand at 2.162 (2) Å (Table 1) from the Cu atom. The N1—Cu1···Cu1' angle is 171.10 (6)° and the dihedral angle between plane through atoms Cu1, O1, O2, C1, Cu1', O1', O2', C1' and the plane through Cu1, O3, O4, C9, Cu1', O3', O4' and C9' atoms is 89.91 (9)°. The dihedral angles between the planar carboxylate groups [(O1/O2/C1) and (O3/O4/C9)] and the adjacent benzene rings A (C2—C7) and B (C10—C15) are 7.88 (19) and 9.68 (10) °, respectively, while that between rings A and B is A/B = 85.90 (9)°. Ring C (N1/C17—C21) is oriented with respect to rings A and B at dihedral angles A/C = 8.59 (7) and B/C = 83.89 (9) °.

In the crystal structure, intermolecular N—H···O hydrogen bonds (Table 2) link the molecules into a three-dimensional network, in which they may be effective in the stabilization of the structure. The π–π contacts between the benzene rings and benzene and pyridine rings, Cg1—Cg1^i^ and Cg3—Cg1^ii^, [symmetry codes (i): 1 - x, -y, 1 - z; (ii) x, y, z - 1, where Cg1 and Cg3 are centroids of the rings A (C2—C7) and C (N1/C17—C21)] may further stabilize the structure, with centroid-centroid distances of 3.563 (2) and 3.484 (2) Å, respectively.

### Experimental

The title compound was prepared by the reaction of CuSO_4_.5H_2_O (1.25 g, 5 mmol) in H_2_O (50 ml) and isonicotinamide (1.22 g, 10 mmol) in H_2_O (20 ml) with sodium 4-methylbenzoate (1.58 g, 10 mmol) in H_2_O (150 ml). The mixture was filtered and set aside to crystallize at ambient temperature for one week, giving green single crystals.

### Refinement

Atoms H2A and H2B (for NH_2_) were located in a difference Fourier map and refined isotropically. The remaining H atoms were positioned geometrically with C—H = 0.95 and 0.98 Å, for aromatic and methyl H atoms, respectively, and constrained to ride on their parent atoms, with U_iso_(H) = xU_eq_(C), where x = 1.5 for methyl H and x = 1.2 for aromatic H atoms.

### Crystal data

**Table:**

[Cu_2_(C_8_H_7_O_2_)_4_(C_6_H_6_N_2_O)_2_]	*F*(000) = 940
*M**_r_* = 911.88	*D*_x_ = 1.467 Mg m^−^^3^
Monoclinic, *P*2_1_/*c*	Mo *K*α radiation, λ = 0.71073 Å
Hall symbol: -P 2ybc	Cell parameters from 3119 reflections
*a* = 11.2305 (2) Å	θ = 2.5–25.4°
*b* = 23.4691 (4) Å	µ = 1.10 mm^−^^1^
*c* = 8.0087 (1) Å	*T* = 101 K
β = 102.128 (1)°	Block, green
*V* = 2063.74 (6) Å^3^	0.30 × 0.24 × 0.14 mm
*Z* = 2	

### Data collection

**Table:**

Bruker Kappa APEXII CCD area-detector diffractometer	5101 independent reflections
Radiation source: fine-focus sealed tube	3629 reflections with *I* > 2σ(*I*)
graphite	*R*_int_ = 0.062
φ and ω scans	θ_max_ = 28.3°, θ_min_ = 1.7°
Absorption correction: multi-scan (*SADABS*; Bruker, 2005)	*h* = −14→11
*T*_min_ = 0.735, *T*_max_ = 0.862	*k* = −31→31
20056 measured reflections	*l* = −10→10

### Refinement

**Table:**

Refinement on *F*^2^	Primary atom site location: structure-invariant direct methods
Least-squares matrix: full	Secondary atom site location: difference Fourier map
*R*[*F*^2^ > 2σ(*F*^2^)] = 0.043	Hydrogen site location: inferred from neighbouring sites
*wR*(*F*^2^) = 0.087	H atoms treated by a mixture of independent and constrained refinement
*S* = 1.01	*w* = 1/[σ^2^(*F*_o_^2^) + (0.0224*P*)^2^ + 2.5778*P*] where *P* = (*F*_o_^2^ + 2*F*_c_^2^)/3
5101 reflections	(Δ/σ)_max_ = 0.001
281 parameters	Δρ_max_ = 0.47 e Å^−^^3^
0 restraints	Δρ_min_ = −0.70 e Å^−^^3^

### Special details

**Table:**

Geometry. All esds (except the esd in the dihedral angle between two l.s. planes) are estimated using the full covariance matrix. The cell esds are taken into account individually in the estimation of esds in distances, angles and torsion angles; correlations between esds in cell parameters are only used when they are defined by crystal symmetry. An approximate (isotropic) treatment of cell esds is used for estimating esds involving l.s. planes.
Refinement. Refinement of *F*^2^ against ALL reflections. The weighted *R*-factor wR and goodness of fit *S* are based on *F*^2^, conventional *R*-factors *R* are based on *F*, with *F* set to zero for negative *F*^2^. The threshold expression of *F*^2^ > σ(*F*^2^) is used only for calculating *R*-factors(gt) etc. and is not relevant to the choice of reflections for refinement. *R*-factors based on *F*^2^ are statistically about twice as large as those based on *F*, and *R*- factors based on ALL data will be even larger.

### Fractional atomic coordinates and isotropic or equivalent isotropic displacement parameters (Å^2^)

**Table:**

	*x*	*y*	*z*	*U*_iso_*/*U*_eq_	
Cu1	0.95060 (3)	0.966319 (13)	1.09852 (4)	0.01099 (9)	
O1	0.81764 (18)	0.95045 (8)	0.9002 (2)	0.0191 (4)	
O2	0.90275 (17)	1.00855 (8)	0.7371 (2)	0.0168 (4)	
O3	1.04702 (19)	0.90818 (8)	1.0089 (2)	0.0203 (4)	
O4	0.86037 (17)	1.03478 (8)	1.1481 (2)	0.0180 (4)	
O5	0.70039 (18)	0.75397 (8)	1.5592 (2)	0.0183 (4)	
N1	0.8976 (2)	0.90548 (9)	1.2719 (3)	0.0139 (5)	
N2	0.7819 (3)	0.81071 (11)	1.7825 (3)	0.0200 (6)	
H2A	0.752 (3)	0.7887 (13)	1.854 (4)	0.022 (8)*	
H2B	0.818 (3)	0.8419 (16)	1.818 (4)	0.047 (12)*	
C1	0.8203 (3)	0.97549 (10)	0.7606 (3)	0.0130 (5)	
C2	0.7165 (2)	0.96483 (11)	0.6131 (3)	0.0122 (5)	
C3	0.6147 (2)	0.93399 (11)	0.6322 (3)	0.0138 (6)	
H3	0.6095	0.9196	0.7413	0.017*	
C4	0.5205 (3)	0.92415 (11)	0.4928 (3)	0.0148 (6)	
H4	0.4508	0.9034	0.5077	0.018*	
C5	0.5265 (3)	0.94428 (11)	0.3308 (3)	0.0147 (6)	
C6	0.6286 (3)	0.97476 (11)	0.3131 (3)	0.0159 (6)	
H6	0.6344	0.9885	0.2035	0.019*	
C7	0.7225 (2)	0.98563 (10)	0.4518 (3)	0.0128 (5)	
H7	0.7911	1.0072	0.4371	0.015*	
C8	0.4259 (3)	0.93213 (12)	0.1789 (3)	0.0221 (6)	
H8A	0.3516	0.9219	0.2179	0.033*	
H8B	0.4498	0.9005	0.1132	0.033*	
H8C	0.4106	0.9661	0.1064	0.033*	
C9	1.1213 (2)	0.91754 (11)	0.9141 (3)	0.0133 (5)	
C10	1.1926 (3)	0.86716 (11)	0.8745 (3)	0.0142 (6)	
C11	1.1638 (3)	0.81263 (11)	0.9227 (3)	0.0188 (6)	
H11	1.0971	0.8075	0.9770	0.023*	
C12	1.2313 (3)	0.76602 (12)	0.8923 (3)	0.0224 (7)	
H12	1.2090	0.7291	0.9232	0.027*	
C13	1.3314 (3)	0.77232 (12)	0.8172 (3)	0.0206 (6)	
C14	1.3589 (3)	0.82663 (12)	0.7674 (3)	0.0203 (6)	
H14	1.4264	0.8318	0.7146	0.024*	
C15	1.2897 (3)	0.87351 (11)	0.7932 (3)	0.0176 (6)	
H15	1.3087	0.9101	0.7552	0.021*	
C16	1.4105 (3)	0.72204 (13)	0.7957 (4)	0.0284 (7)	
H16A	1.3606	0.6874	0.7780	0.043*	
H16B	1.4748	0.7177	0.8985	0.043*	
H16C	1.4473	0.7283	0.6967	0.043*	
C17	0.7944 (2)	0.87572 (10)	1.2239 (3)	0.0134 (5)	
H17	0.7501	0.8796	1.1096	0.016*	
C18	0.7487 (2)	0.83964 (11)	1.3316 (3)	0.0124 (5)	
H18	0.6757	0.8188	1.2916	0.015*	
C19	0.8121 (2)	0.83446 (10)	1.4999 (3)	0.0127 (5)	
C20	0.9206 (3)	0.86403 (11)	1.5496 (3)	0.0170 (6)	
H20	0.9674	0.8604	1.6626	0.020*	
C21	0.9600 (3)	0.89878 (11)	1.4330 (3)	0.0152 (6)	
H21	1.0346	0.9188	1.4683	0.018*	
C22	0.7601 (3)	0.79620 (11)	1.6172 (3)	0.0142 (6)	

### Atomic displacement parameters (Å^2^)

**Table:**

	*U*^11^	*U*^22^	*U*^33^	*U*^12^	*U*^13^	*U*^23^
Cu1	0.01279 (18)	0.01089 (15)	0.00968 (13)	−0.00136 (15)	0.00323 (11)	0.00055 (13)
O1	0.0192 (11)	0.0233 (11)	0.0133 (9)	−0.0080 (9)	0.0000 (8)	0.0042 (7)
O2	0.0154 (11)	0.0202 (10)	0.0137 (8)	−0.0071 (8)	0.0003 (7)	0.0035 (7)
O3	0.0299 (13)	0.0129 (9)	0.0236 (10)	0.0023 (9)	0.0177 (9)	0.0009 (8)
O4	0.0208 (11)	0.0142 (9)	0.0221 (9)	0.0023 (9)	0.0116 (8)	0.0033 (8)
O5	0.0255 (12)	0.0147 (10)	0.0160 (9)	−0.0046 (9)	0.0077 (8)	0.0016 (7)
N1	0.0156 (13)	0.0140 (11)	0.0130 (10)	0.0012 (10)	0.0050 (9)	0.0011 (8)
N2	0.0348 (17)	0.0132 (12)	0.0140 (11)	−0.0057 (12)	0.0094 (11)	0.0010 (9)
C1	0.0174 (15)	0.0085 (13)	0.0143 (11)	0.0016 (11)	0.0058 (10)	−0.0026 (9)
C2	0.0137 (14)	0.0096 (11)	0.0128 (11)	0.0027 (11)	0.0018 (9)	−0.0023 (10)
C3	0.0153 (16)	0.0133 (13)	0.0141 (12)	0.0024 (11)	0.0061 (10)	0.0007 (10)
C4	0.0127 (15)	0.0136 (13)	0.0179 (12)	−0.0008 (11)	0.0028 (11)	−0.0016 (10)
C5	0.0161 (16)	0.0110 (12)	0.0162 (12)	0.0039 (11)	0.0011 (10)	−0.0026 (10)
C6	0.0218 (16)	0.0124 (13)	0.0138 (11)	0.0015 (11)	0.0045 (11)	0.0006 (10)
C7	0.0144 (15)	0.0103 (12)	0.0150 (12)	0.0002 (11)	0.0062 (10)	0.0001 (9)
C8	0.0226 (18)	0.0209 (15)	0.0198 (13)	−0.0015 (13)	−0.0026 (12)	−0.0029 (11)
C9	0.0125 (15)	0.0159 (13)	0.0099 (11)	−0.0029 (11)	−0.0013 (10)	−0.0023 (9)
C10	0.0161 (16)	0.0154 (13)	0.0104 (11)	0.0002 (11)	0.0013 (10)	−0.0003 (10)
C11	0.0192 (17)	0.0180 (14)	0.0195 (13)	−0.0011 (12)	0.0049 (11)	0.0025 (11)
C12	0.0265 (19)	0.0149 (14)	0.0241 (14)	0.0034 (13)	0.0017 (12)	0.0022 (11)
C13	0.0245 (18)	0.0209 (15)	0.0149 (13)	0.0078 (13)	0.0009 (11)	−0.0017 (11)
C14	0.0202 (17)	0.0247 (16)	0.0171 (13)	0.0041 (13)	0.0063 (11)	−0.0008 (11)
C15	0.0222 (17)	0.0136 (13)	0.0160 (12)	0.0004 (12)	0.0016 (11)	0.0003 (10)
C16	0.036 (2)	0.0219 (16)	0.0264 (15)	0.0131 (14)	0.0043 (14)	0.0000 (12)
C17	0.0183 (16)	0.0113 (12)	0.0110 (11)	0.0004 (11)	0.0036 (10)	−0.0014 (9)
C18	0.0138 (15)	0.0112 (12)	0.0130 (11)	−0.0006 (11)	0.0049 (10)	−0.0026 (10)
C19	0.0177 (16)	0.0093 (12)	0.0127 (11)	0.0007 (11)	0.0065 (10)	−0.0005 (9)
C20	0.0202 (17)	0.0189 (14)	0.0108 (11)	−0.0003 (12)	0.0003 (10)	0.0026 (10)
C21	0.0140 (15)	0.0152 (13)	0.0160 (12)	−0.0036 (11)	0.0026 (10)	0.0002 (10)
C22	0.0186 (16)	0.0134 (13)	0.0118 (12)	0.0009 (12)	0.0060 (11)	0.0033 (10)

### Geometric parameters (Å, °)

**Table:**

Cu1—Cu1^i^	2.6375 (6)	C8—H8A	0.9800
Cu1—O1	1.9733 (18)	C8—H8B	0.9800
Cu1—O2^i^	1.9703 (18)	C8—H8C	0.9800
Cu1—O3	1.9687 (18)	C9—O4^i^	1.259 (3)
Cu1—O4	1.9836 (18)	C9—C10	1.499 (4)
Cu1—N1	2.161 (2)	C10—C11	1.394 (4)
O1—C1	1.269 (3)	C10—C15	1.390 (4)
O2—Cu1^i^	1.9703 (18)	C11—C12	1.381 (4)
O2—C1	1.252 (3)	C11—H11	0.9500
O3—C9	1.259 (3)	C12—C13	1.389 (4)
O4—C9^i^	1.259 (3)	C12—H12	0.9500
O5—C22	1.232 (3)	C13—C14	1.389 (4)
N1—C17	1.338 (3)	C13—C16	1.508 (4)
N1—C21	1.342 (3)	C14—C15	1.387 (4)
N2—C22	1.339 (3)	C14—H14	0.9500
N2—H2A	0.89 (3)	C15—H15	0.9500
N2—H2B	0.85 (4)	C16—H16A	0.9800
C1—C2	1.497 (3)	C16—H16B	0.9800
C2—C3	1.388 (4)	C16—H16C	0.9800
C2—C7	1.397 (3)	C17—C18	1.382 (3)
C3—C4	1.387 (4)	C17—H17	0.9500
C3—H3	0.9500	C18—C19	1.391 (3)
C4—C5	1.395 (3)	C18—H18	0.9500
C4—H4	0.9500	C19—C20	1.387 (4)
C5—C6	1.384 (4)	C19—C22	1.503 (3)
C5—C8	1.503 (4)	C20—C21	1.381 (3)
C6—C7	1.385 (4)	C20—H20	0.9500
C6—H6	0.9500	C21—H21	0.9500
C7—H7	0.9500		
			
O1—Cu1—Cu1^i^	88.51 (5)	H8A—C8—H8B	109.5
O1—Cu1—O4	88.96 (8)	H8A—C8—H8C	109.5
O1—Cu1—N1	97.37 (8)	H8B—C8—H8C	109.5
O2^i^—Cu1—Cu1^i^	79.79 (5)	O3—C9—O4^i^	125.3 (2)
O2^i^—Cu1—O1	168.28 (7)	O3—C9—C10	116.2 (2)
O2^i^—Cu1—O4	90.82 (8)	O4^i^—C9—C10	118.6 (2)
O2^i^—Cu1—N1	94.19 (8)	C11—C10—C9	120.0 (2)
O3—Cu1—Cu1^i^	82.21 (5)	C15—C10—C9	121.4 (2)
O3—Cu1—O1	87.53 (8)	C15—C10—C11	118.6 (3)
O3—Cu1—O2^i^	90.25 (8)	C10—C11—H11	119.6
O3—Cu1—O4	167.82 (7)	C12—C11—C10	120.7 (3)
O3—Cu1—N1	91.34 (8)	C12—C11—H11	119.6
O4—Cu1—Cu1^i^	86.04 (5)	C11—C12—C13	121.1 (3)
O4—Cu1—N1	100.68 (8)	C11—C12—H12	119.5
N1—Cu1—Cu1^i^	171.10 (6)	C13—C12—H12	119.5
C1—O1—Cu1	117.87 (17)	C12—C13—C16	121.0 (3)
C1—O2—Cu1^i^	128.72 (16)	C14—C13—C12	118.1 (3)
C9—O3—Cu1	125.69 (17)	C14—C13—C16	120.9 (3)
C9^i^—O4—Cu1	120.50 (17)	C13—C14—H14	119.3
C17—N1—Cu1	119.94 (16)	C15—C14—C13	121.3 (3)
C17—N1—C21	117.5 (2)	C15—C14—H14	119.3
C21—N1—Cu1	122.42 (18)	C10—C15—H15	119.9
C22—N2—H2A	118 (2)	C14—C15—C10	120.2 (3)
C22—N2—H2B	121 (2)	C14—C15—H15	119.9
H2A—N2—H2B	120 (3)	C13—C16—H16A	109.5
O1—C1—C2	117.3 (2)	C13—C16—H16B	109.5
O2—C1—O1	125.1 (2)	C13—C16—H16C	109.5
O2—C1—C2	117.6 (2)	H16A—C16—H16B	109.5
C3—C2—C1	121.6 (2)	H16A—C16—H16C	109.5
C3—C2—C7	119.1 (2)	H16B—C16—H16C	109.5
C7—C2—C1	119.4 (2)	N1—C17—C18	123.6 (2)
C2—C3—H3	119.9	N1—C17—H17	118.2
C4—C3—C2	120.3 (2)	C18—C17—H17	118.2
C4—C3—H3	119.9	C17—C18—C19	118.5 (2)
C3—C4—C5	121.0 (3)	C17—C18—H18	120.7
C3—C4—H4	119.5	C19—C18—H18	120.7
C5—C4—H4	119.5	C18—C19—C22	118.1 (2)
C4—C5—C8	120.9 (3)	C20—C19—C18	118.3 (2)
C6—C5—C4	118.2 (2)	C20—C19—C22	123.6 (2)
C6—C5—C8	120.9 (2)	C19—C20—H20	120.4
C5—C6—C7	121.4 (2)	C21—C20—C19	119.3 (2)
C5—C6—H6	119.3	C21—C20—H20	120.4
C7—C6—H6	119.3	N1—C21—C20	122.8 (3)
C2—C7—H7	120.0	N1—C21—H21	118.6
C6—C7—C2	120.0 (2)	C20—C21—H21	118.6
C6—C7—H7	120.0	O5—C22—N2	123.4 (2)
C5—C8—H8A	109.5	O5—C22—C19	119.8 (2)
C5—C8—H8B	109.5	N2—C22—C19	116.9 (2)
C5—C8—H8C	109.5		
			
Cu1^i^—Cu1—O1—C1	−1.45 (18)	O2—C1—C2—C7	8.2 (4)
O2^i^—Cu1—O1—C1	−4.4 (5)	C1—C2—C3—C4	−179.2 (2)
O3—Cu1—O1—C1	−83.72 (19)	C7—C2—C3—C4	−0.1 (4)
O4—Cu1—O1—C1	84.61 (19)	C1—C2—C7—C6	178.2 (2)
N1—Cu1—O1—C1	−174.75 (19)	C3—C2—C7—C6	−0.9 (4)
Cu1^i^—Cu1—O3—C9	5.3 (2)	C2—C3—C4—C5	0.8 (4)
O1—Cu1—O3—C9	94.1 (2)	C3—C4—C5—C6	−0.5 (4)
O2^i^—Cu1—O3—C9	−74.4 (2)	C3—C4—C5—C8	178.2 (2)
O4—Cu1—O3—C9	20.7 (5)	C4—C5—C6—C7	−0.5 (4)
N1—Cu1—O3—C9	−168.6 (2)	C8—C5—C6—C7	−179.2 (2)
Cu1^i^—Cu1—O4—C9^i^	−2.61 (18)	C5—C6—C7—C2	1.2 (4)
O1—Cu1—O4—C9^i^	−91.19 (19)	O3—C9—C10—C11	8.4 (4)
O2^i^—Cu1—O4—C9^i^	77.09 (19)	O3—C9—C10—C15	−170.2 (2)
O3—Cu1—O4—C9^i^	−17.9 (5)	O4^i^—C9—C10—C11	−171.8 (2)
N1—Cu1—O4—C9^i^	171.51 (19)	O4^i^—C9—C10—C15	9.6 (4)
O1—Cu1—N1—C17	−4.4 (2)	C9—C10—C11—C12	−177.8 (2)
O1—Cu1—N1—C21	−179.6 (2)	C15—C10—C11—C12	0.8 (4)
O2^i^—Cu1—N1—C17	177.58 (19)	C9—C10—C15—C14	176.1 (2)
O2^i^—Cu1—N1—C21	2.4 (2)	C11—C10—C15—C14	−2.6 (4)
O3—Cu1—N1—C17	−92.1 (2)	C10—C11—C12—C13	1.7 (4)
O3—Cu1—N1—C21	92.7 (2)	C11—C12—C13—C14	−2.5 (4)
O4—Cu1—N1—C17	85.95 (19)	C11—C12—C13—C16	175.5 (3)
O4—Cu1—N1—C21	−89.3 (2)	C12—C13—C14—C15	0.8 (4)
Cu1—O1—C1—O2	2.0 (3)	C16—C13—C14—C15	−177.3 (3)
Cu1—O1—C1—C2	−177.69 (16)	C13—C14—C15—C10	1.8 (4)
Cu1^i^—O2—C1—O1	−1.3 (4)	N1—C17—C18—C19	1.0 (4)
Cu1^i^—O2—C1—C2	178.39 (16)	C17—C18—C19—C20	−2.6 (4)
Cu1—O3—C9—O4^i^	−5.1 (4)	C17—C18—C19—C22	178.6 (2)
Cu1—O3—C9—C10	174.71 (16)	C18—C19—C20—C21	2.1 (4)
Cu1—N1—C17—C18	−174.35 (19)	C22—C19—C20—C21	−179.1 (2)
C21—N1—C17—C18	1.1 (4)	C18—C19—C22—O5	30.9 (4)
Cu1—N1—C21—C20	173.7 (2)	C18—C19—C22—N2	−148.8 (3)
C17—N1—C21—C20	−1.6 (4)	C20—C19—C22—O5	−147.9 (3)
O1—C1—C2—C3	7.0 (4)	C20—C19—C22—N2	32.4 (4)
O1—C1—C2—C7	−172.1 (2)	C19—C20—C21—N1	0.1 (4)
O2—C1—C2—C3	−172.7 (2)		

Symmetry codes: (i) −*x*+2, −*y*+2, −*z*+2.

### Hydrogen-bond geometry (Å, °)

**Table:**

*D*—H···*A*	*D*—H	H···*A*	*D*···*A*	*D*—H···*A*
N2—H2A···O5^ii^	0.89 (3)	2.11 (3)	2.984 (3)	169 (3)

Symmetry codes: (ii) *x*, −*y*+3/2, *z*+1/2.
